# Surface Plasmon Resonance Sensor Based on Fe_2_O_3_/Au for Alcohol Concentration Detection

**DOI:** 10.3390/s24144477

**Published:** 2024-07-11

**Authors:** Junyi Wang, Yanpei Xu, Yutong Song, Qi Wang

**Affiliations:** College of Sciences, Northeastern University, Shenyang 110819, China; 20221741@stu.neu.edu.cn (J.W.); xuyanpei@stumail.neu.edu.cn (Y.X.); songyt@mail.neu.edu.cn (Y.S.)

**Keywords:** SPR, Fe_2_O_3_, refractive index sensing, alcohol concentration detection, FEM simulation

## Abstract

Hematite (α-Fe_2_O_3_) is widely used in sensor sensitization due to its excellent optical properties. In this study, we present a sensitivity-enhanced surface plasmon resonance alcohol sensor based on Fe_2_O_3_/Au. We describe the fabrication process of the sensor and characterize its structure. We conduct performance testing on sensors coated multiple times and use solutions with the same gradient of refractive indices as the sensing medium. Within the refractive index range of 1.3335–1.3635, the sensor that was coated twice achieved the highest sensitivity, reaching 2933.2 nm/RIU. This represents a 30.26% enhancement in sensitivity compared to a sensor with a pure gold monolayer film structure. Additionally, we demonstrated the application of this sensor in alcohol concentration detection by testing the alcohol content of common beverages, showing excellent agreement with theoretical values and highlighting the sensor’s potential in food testing.

## 1. Introduction

Metal surfaces have abundant free electrons, which, under the influence of incident light, can oscillate collectively. This phenomenon is known as surface plasmon waves (SPWs). When the incident light resonates with SPWs under specific conditions, it results in a sharp decline in the intensity of the reflected light, a phenomenon referred to as surface plasmon resonance (SPR) [1,2]. The wave vector of SPWs is highly sensitive to the refractive index of dielectrics; thus, changes in the refractive index can lead to a corresponding shift in the SPR absorption peak. This makes it capable of detecting changes in the refractive index of dielectrics [3]. In recent years, SPR has been employed in the creation of biological and chemical sensors [4,5] and has been widely applied in various fields such as biology, pharmaceuticals, and environmental safety, due to its low cost and high sensitivity in characterization [6,7,8].

Developing a highly sensitive SPR sensor is of great significance. In addition, the detection of low-concentration target molecules using SPR sensors has been greatly hindered by the minimal changes in dielectric properties at the metal/dielectric interface and the poor absorption capacity of metals [9]. Therefore, there is an urgent need for a simple and reliable method to enhance SPR sensors.

In fields such as food processing, medical diagnosis, and public safety, alcohol is a commonly encountered chemical substance [10,11]. The measurement of alcohol concentration is crucial for ensuring people’s health and safety [12]. Therefore, the development of a rapid sensor with high sensitivity and precise measurement of alcohol concentration holds significant application prospects. The SPR sensor, owing to its excellent detection performance and label-free nature, has emerged as a highly promising sensor for alcohol concentration [13]. Furthermore, the high sensitivity of the SPR sensor can be harnessed to enhance the precision and accuracy of alcohol concentration detection [14]. Thus, SPR technology stands as a highly promising technique for alcohol concentration sensing.

In 2020, Zhang et al. proposed an optical fiber sensor combining multi-mode interference (MMI) and surface plasmon resonance structures, achieving the highest refractive index sensitivity of 2061.6 nm/RIU [15]. The feasibility of the sensor in liquid alcohol was validated. In 2021, Gao et al. designed a single-layer graphene/HMM/D-shaped plastic optical fiber (G/HMM/D-POF) SPR sensor for detecting ethanol solution, attaining a sensitivity of 5166.7 nm/RIU [16]. Wang et al. designed and analyzed an SPR temperature sensor based on a connected tubular resonance fiber (CTF), where alcohol was injected into the core of the CTF as the sensing medium fabricated by vapor deposition [17]. In 2022, Xu et al. introduced an SPR sensor based on ZnO nanoflowers/Au, achieving a sensitivity of 1.27 nm/% for alcohol concentration [18].

Gold is widely used in the fabrication of SPR sensors due to its unique optical, chemical, and catalytic properties [19,20,21]. Nanoscale gold films can effectively enhance the sensitivity of refractive index sensing [22]. To further improve the sensitivity and selectivity of SPR sensors, modifying the gold film surface with sensing materials has become a choice. In recent years, many studies have successfully enhanced the sensitivity of SPR sensors using modification layers [18,23]. Nanoscale iron oxide is a sensitive material. Nano α-Fe_2_O_3_ particles possess unique optical properties and exhibit good absorption capabilities around 550 nm [24]; thus, they have been extensively studied as a novel photocatalytic material [25,26,27,28]. Additionally, due to their chemical structure, nano α-Fe_2_O_3_ particles have good adsorption properties [29]. Therefore, modifying the gold film with a nano α-Fe_2_O_3_ coating can improve the sensitivity of SPR refractive index sensors.

This study enhances the sensitivity of gold film-based SPR refractive index sensors using nano α-Fe_2_O_3_. By spin-coating different amounts of nano α-Fe_2_O_3_ films on the gold film and comparing them with pure gold film structures, the sensors with one to three cycles of spin-coating exhibited sensitivities of 2550.45 nm, 2933.2 nm, and 2522.16 nm, respectively, showing a significant improvement over the uncoated SPR sensors. Finally, this study utilized the nano α-Fe_2_O_3_-Au SPR refractive index sensor to measure the alcohol content of alcoholic beverages, demonstrating the potential applications of this sensor in industries such as food processing and chemical engineering.

## 2. Theoretical Foundation

SPR is an optical phenomenon that arises from the interaction of incident light with the surface plasmon resonances on a metal surface. It is employed in biosensing and chemical analysis. The generation of SPR can be described by considering the wave vector of the evanescent wave and the wave vector mismatch of the surface plasmon wave.

An evanescent wave, which appears during total internal reflection, is expressed through its electric field wave vector, $k_{x}$, as given in the following equation [30]:

(1) $$k_{x}=\frac{\omega }{c}\sqrt{\varepsilon_{0}}\sin\theta .$$

Surface plasmon waves arise from the collective oscillations of electrons within a metal. The wave vector for surface plasmons, represented by $k_{sp}$, is outlined in the following equation [31]:

(2) $$k_{sp}=\frac{\omega }{c}\sqrt{\frac{\varepsilon_{1}\varepsilon_{2}}{\varepsilon_{1}+\varepsilon_{2}}}.$$

When $k_{x}$ equals $k_{sp}$, the energy from the evanescent wave is transferred to the surface plasmon wave. This transfer is essential for the excitation of SPR.

In the equation referenced, ω symbolizes the angular frequency of the incident light, θ represents the angle of incidence, *c* is the speed of light, and $\varepsilon_{0}$ is the dielectric constant of the prism. Additionally, $\varepsilon_{1}$ is identified as the dielectric constant of the metal film, and $\varepsilon_{2}$ is the dielectric constant of the test medium being analyzed. Importantly, the magnitude of the evanescent wave is independent of both the metal film type and the test medium.

This coupling facilitates the oscillation and propagation of light energy as electromagnetic waves between the metal surface and the dielectric, leading to the occurrence of surface plasmon resonance.

## 3. Experimental Section

### 3.1. Materials and Reagents

Ferric chloride (FeCl_3_), hexamethylenetetramine, sodium bicarbonate, anhydrous ethanol (99%), hydrochloric acid, and acetone (99.5%) were all purchased from Aladdin Company (Shanghai, China). The gold target material was acquired from Shenyang Kejing Automation Equipment Co., Ltd. (Shenyang, China). All chemical reagents used were of analytical grade and could be used without further purification.

### 3.2. Sensor Preparation

**Figure 1:**
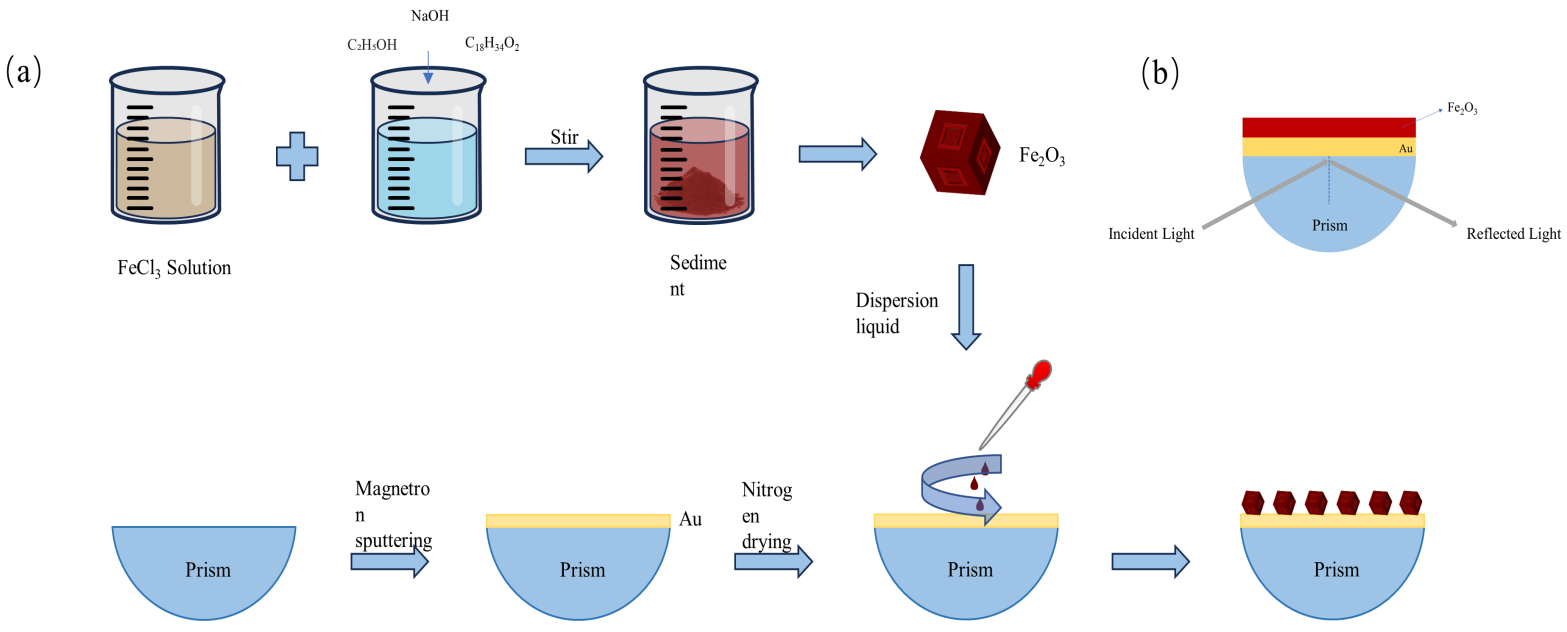
(**a**) Schematic diagram of the sensor fabrication process. (**b**) Schematic diagram of the sensor structure.

The fabrication process of the sensor is shown in Figure 1a. Firstly, a gold film was deposited on the prism. The prism was ultrasonically cleaned in a mixture of acetone and deionized water, then dried in an oven at 60 °C. A magnetron sputtering coater (GSL-1100X-SPC-16 M, Shenyang Kejing Automation Equipment Co., Ltd., Shenyang, China) was used to deposit a 50 nm gold film on the surface of the prism under vacuum conditions (pressure less than 1 × 10^−1^ Pa) and a current of about 15 mA.

Next, nano α-Fe_2_O_3_ was synthesized as follows: 5.7 mmol of hexamethylenetetramine and 1.48 mmol of ferric chloride were dissolved in 50 mL of deionized water. Dilute hydrochloric acid was slowly added to the solution to adjust the pH to 1.8. The solution was then transferred to a reactor lined and preheated to 130 °C in an oven for 20 h of heating. After heating, the reaction was quenched in a cold water bath, and the liquid was centrifuged at 4900 rpm for 10 min. The supernatant was discarded, and the precipitate was resuspended in deionized water using an ultrasonic cleaner, followed by centrifugal washing repeated three times. The product was then dried at 65 °C for 12 h to obtain the final product.

Finally, a dispersion was prepared, and the nano α-Fe_2_O_3_ was dispersed on the Au film. Then, 2 mg of α-Fe_2_O_3_ was dispersed in 8 mL of deionized water. Next, 50 µL of the dispersion was dropped onto the center of the gold film surface, then spread evenly using a spin-coating method. The spin-coating parameters were 1200 rpm for 30 s. The deposition of varying quantities of α-Fe_2_O_3_ on a gold film surface can be achieved by manipulating the number of spin-coating cycles.

We used an Abbe refractometer and a pipette to prepare 200 mL of solution for each refractive index (1.3335–1.3635, with intervals of 0.0075), one at a time. For solutions that did not reach the target refractive index, we added ethanol, and for solutions that exceeded the target refractive index, we diluted them until the solution reached the target refractive index. Additionally, the solutions we prepared were stored in sealed containers and only opened when used, ensuring that there was no ethanol evaporation loss.

### 3.3. Experimental Apparatus

The experimental optical path consists sequentially of a fiber-optic tungsten halogen lamp, a polarizer, an aperture diaphragm, a prism, an optical integrating sphere, and a spectrometer. The experiment employs wavelength modulation, using a laser with a broad spectral range (360–2500 nm) as the light source. The incident light is adjusted to p-polarized light by the polarizer and is directed through the aperture diaphragm near the prism to minimize the divergence angle of the light beam. The light beam hits the prism and undergoes total internal reflection at the metal interface (Figure 1b). The reflected light is captured by the integrating sphere and connected to the spectrometer (Flame-T-VIS-NIR, Ocean Insight, Orlando, FL, USA), where spectral data are analyzed by specialized software.

## 4. Results and Discussion

### 4.1. Sensor Performance Measurement

**Figure 2:**
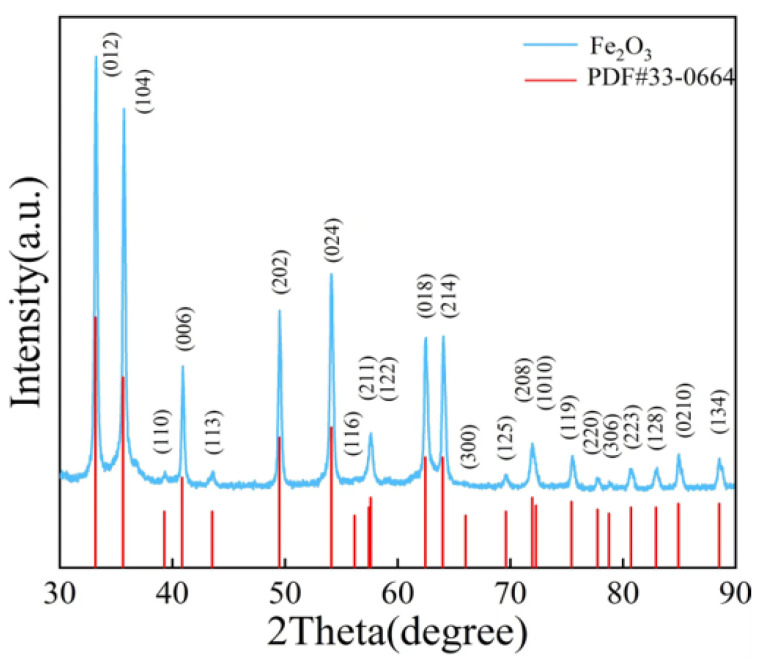
XRD spectrum of the synthesized Fe_2_O_3_ compared with the standard spectrum.

**Figure 3:**
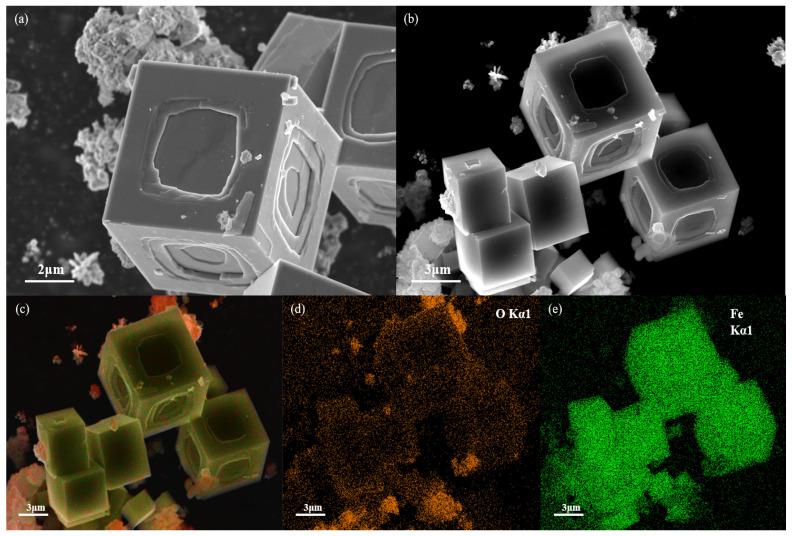
(**a**,**b**) The SEM images of the synthesized Fe_2_O_3_; (**c**) electron micrograph of the synthesized Fe_2_O_3_; (**d**,**e**) the distributions of O, Fe.

Figure 2 displays the XRD image of nano α-Fe_2_O_3_ (in blue) alongside the image of the α-Fe_2_O_3_ standard card (in red, PDF#33-0664), showing that the XRD peaks of the nano α-Fe_2_O_3_ align well with those of the standard card. Figure 3a,b present SEM images of nano α-Fe_2_O_3_ from two different perspectives, revealing that the synthesized nano α-Fe_2_O_3_ has a hexagonal structure. To further verify the elemental composition of the synthesized material, Figure 3c–e display the elemental maps of the synthesis, indicating the presence of oxygen and iron elements, confirming the synthesized product as α-Fe_2_O_3_.

**Figure 4:**
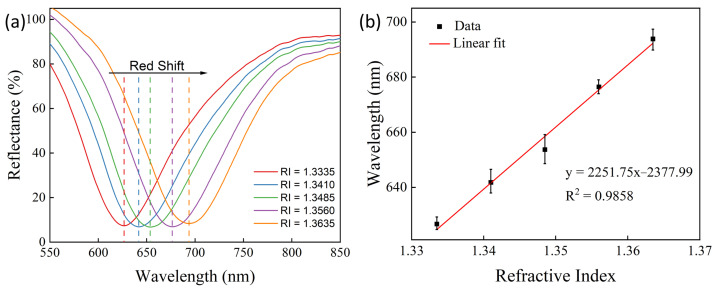
The SPR spectra of the refractive index sensing performance of a device based on a 50 nm Au structure. (**a**) SPR signals for substances with different refractive indices. (**b**) Linear fitting of the sensing signals.

**Figure 5:**
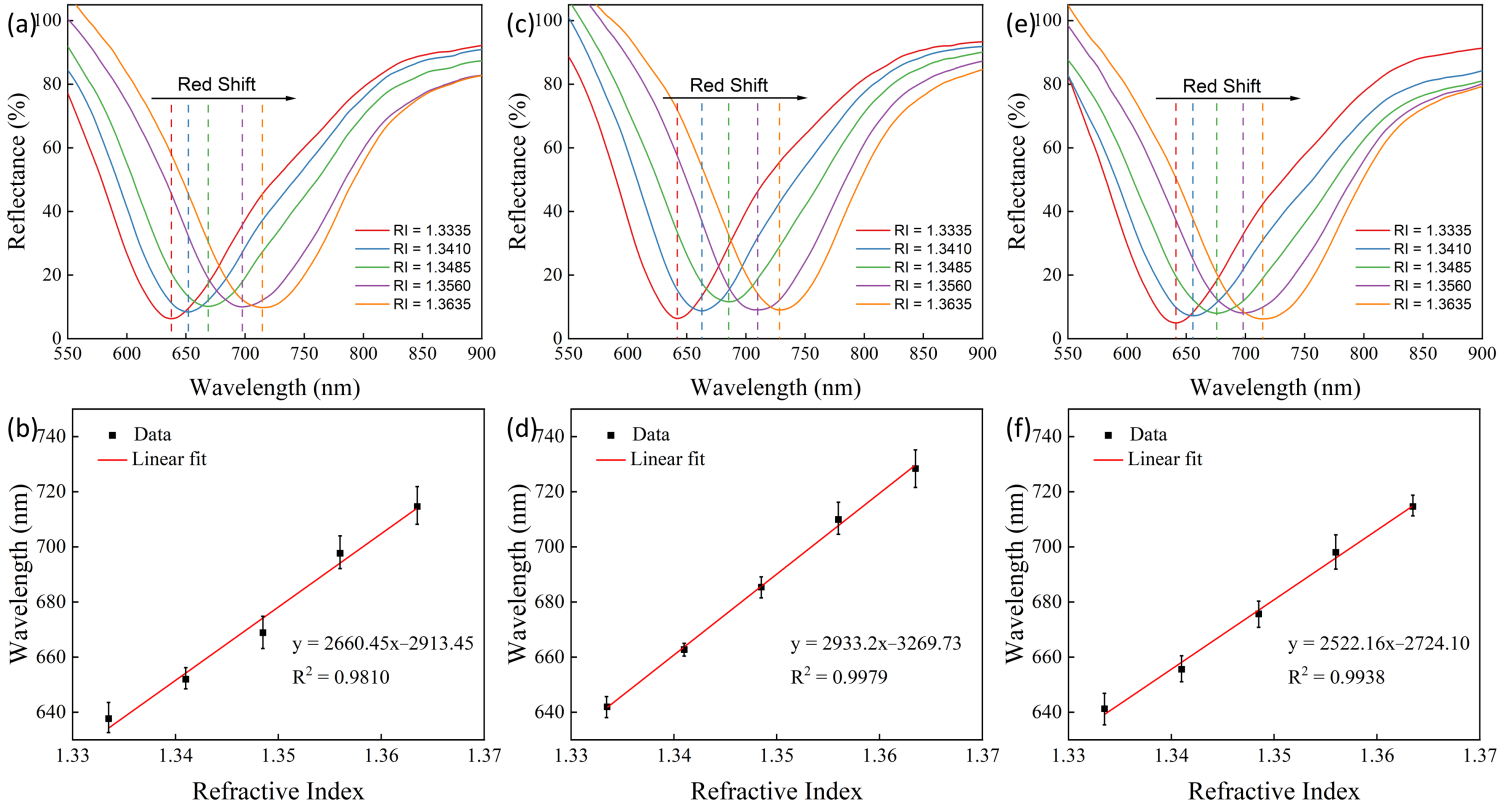
Refractive index testing performance diagram of the sensor with a nano Fe_2_O_3_/Au structure. (**a**) Response signals of Fe_2_O_3_/Au sensors to different refractive index media under a single spin-coating cycle. (**b**) Linear fitting of different refractive indices for the Fe_2_O_3_/Au sensors under a single spin-coating cycle. (**c**) Response signals of Fe_2_O_3_/Au sensors to different refractive index media under two spin-coating cycles. (**d**) Linear fitting of different refractive indices for the Fe_2_O_3_/Au sensors under two spin-coating cycles. (**e**) Response signals of Fe_2_O_3_/Au sensors to different refractive index media under three spin-coating cycles. (**f**) Linear fitting of different refractive indices for the Fe_2_O_3_/Au sensors under three spin-coating cycles.

The experiment was conducted at 25 °C. Figure 4a and Figure 5a,c,e display the SPR signals from a prism device for different refractive indices. The testing temperature was approximately 20 °C, and as the refractive index increased, a redshift occurred in the SPR absorption trough. The relationship between the SPR absorption trough wavelengths and the corresponding refractive indices was plotted as fitting curves, as shown in Figure 4b and Figure 5b,d,f. The devices corresponding to Figure 4a and Figure 5a,c,e are labeled as Devices 1, 2, 3, and 4, corresponding respectively to the prism devices coated with an Au film without nano α-Fe_2_O_3_, with one spin-coating cycle of nano α-Fe_2_O_3_ film, with two spin-coating cycles of nano α-Fe_2_O_3_ film, and with three spin-coating cycles of nano α-Fe_2_O_3_ film. For Device 1, the absorption trough shifted from 666.70 nm to 733.83 nm; for Device 2, from 637.69 nm to 714.63 nm; for Device 3, from 641.98 nm to 728.39 nm; and for Device 4, from 641.27 nm to 714.63 nm. It is evident that the presence of α-Fe_2_O_3_ reduced the corresponding wavelength of the absorption trough while increasing the range of the wavelength shift. The greater the shift in the absorption trough wavelength with changes in the refractive index, the stronger the sensitivity of the device to refractive index changes, which is defined by the sensitivity formula:

(3) $$S=\frac{\delta \lambda }{\delta R},$$

where λ represents the wavelength of the SPR absorption trough, and R represents the refractive index. To characterize the sensitivity performance of the sensors, the absorption trough wavelengths and refractive indices were fitted. From Figure 4b and Figure 5b,d,f, the goodness-of-fit for Devices 1 to 4 in the refractive index range from 1.3335 to 1.3635 were 0.9858, 0.9810, 0.9979, and 0.9938, respectively, showing strong linearity for all devices. The slope of the fitted line, which is the sensitivity value, for Devices 1 to 4 were, respectively, 2251.75 nm, 2550.45 nm, 2933.2 nm, and 2522.16 nm. This demonstrates that Devices 2, 3, and 4 showed a sensitivity improvement of 13.27%, 30.26%, and 12.01%, respectively, compared to Device 1, with Devices 3 and 4 also showing an increase in goodness-of-fit by 1.23% and 8.12%, respectively. This indicates that α-Fe_2_O_3_ effectively and significantly enhances the sensitivity of the devices to refractive index measurement, with the device coated with two spin-coating cycles of nano α-Fe_2_O_3_ film exhibiting the highest sensitivity and best performance. The relationship between refractive index (*n*) and concentration (*c*) obtained through least squares fitting is given by the following equation [32]:

(4) n=1.33307+0.4643c.

Sensitivity is a vital measure of performance for SPR sensors. It can be expressed in terms of refractive index sensitivity, represented as $\frac{\Delta \lambda }{\Delta n}$, and concentration sensitivity, denoted as $\frac{\Delta \lambda }{\Delta c}$. Here, Δλ denotes the shift in the resonance wavelength, Δn indicates changes in the refractive index of the medium being sensed, and Δc refers to variations in alcohol concentration. It is important to note that the alcohols used in the study were all ethanol. Table 1 illustrates a comparison of the performance metrics of the sensor in this study against those from other related studies. It is worth noting that, for each of the sensor surfaces (zero to three spin-coating cycles), three replicated sensor surfaces were manufactured, and five independent replicated experiments were conducted to measure SPR responses.

### 4.2. Analysis and Explanation

When the coated device comes into contact with alcohol, ethanol molecules are adsorbed onto the nano/Au film. At this point, the ethanol molecules are ionized by the action of the film surface electrons to form H^+^. These H^+^ ions combine with the electrons on the film surface and are dispersed more quickly through a spillover mechanism [35,36]. Meanwhile, the photo-generated electrons, driven by differences in electron concentration, move to the Au layer and scatter again, further enhancing the SPR efficiency [37].

The intrinsic electrical conductivity of this setup is relatively low, which slows down the charge transport within the material. On the other hand, a deeper absorption depth is required, meaning the nano-modified coating layer cannot be too thin to prevent excessive photons from not being absorbed, nor too thick to avoid rapid electron–hole recombination. Therefore, the thickness of the nano-modified coating layer impacts the performance of the sensor [38]. Additionally, some studies have demonstrated that using conductive precious metals like Au as the substrate material can mitigate the negative effects brought on by low intrinsic conductivity [39].

**Figure 6:**
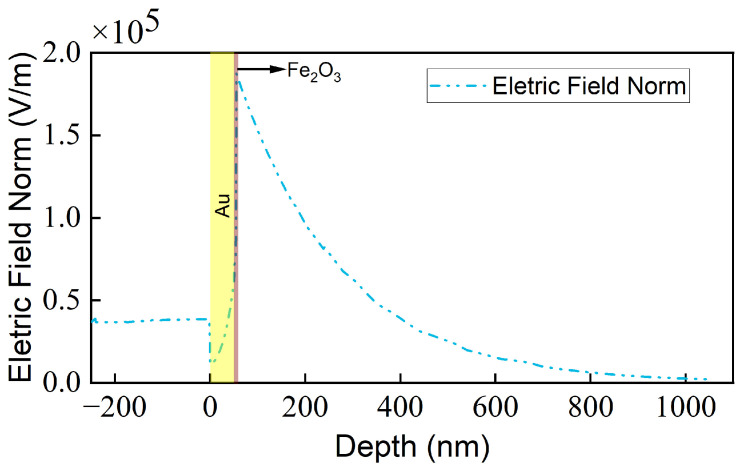
Enhanced electric field mode diagram of the sensor.

More specifically, the introduction of an additional layer of Fe_2_O_3_ with a higher refractive index effectively transforms the sensing layer into a hybrid medium comprising both the sensing medium and Fe_2_O_3_. The corresponding effective refractive index can be expressed by the following equation:

(5) $$n_{eff}=n_{analyte}\times c_{analyte}+n_{Fe_{2}O_{3}}\times c_{Fe_{2}O_{3}},$$

where $c_{analyte}$ and $c_{Fe_{2}O_{3}}$ represent the volume fractions of the sensing medium, and Fe_2_O_3_, $n_{analyte}$ and $n_{Fe_{2}O_{3}}$ denote the refractive index of the analyte in the sensing layer and the Fe_2_O_3_ in the sensing layer, respectively. Additionally, through finite element (FEM) analysis, Figure 6 illustrates the distribution of the electric field mode in alignment with the incident surface normal, demonstrating that the electric field is initially amplified at the gold film and subsequently enhanced within Fe_2_O_3_ before experiencing exponential decay. It is widely accepted that the distance from the interface at which the electric field mode diminishes to 1/e of its peak value denotes the sensing distance, signifying the primary area contributing to the actual sensing effect. As depicted in Figure 6, a 50 nm gold film elevates the electric field mode to 61,500 V/m, while further enhancement to 187,400 V/m is realized in Fe_2_O_3_. This suggests that incorporating a Fe_2_O_3_ coating will markedly expand the sensor’s contribution area, thereby amplifying the sensor’s sensitivity.

**Figure 7:**
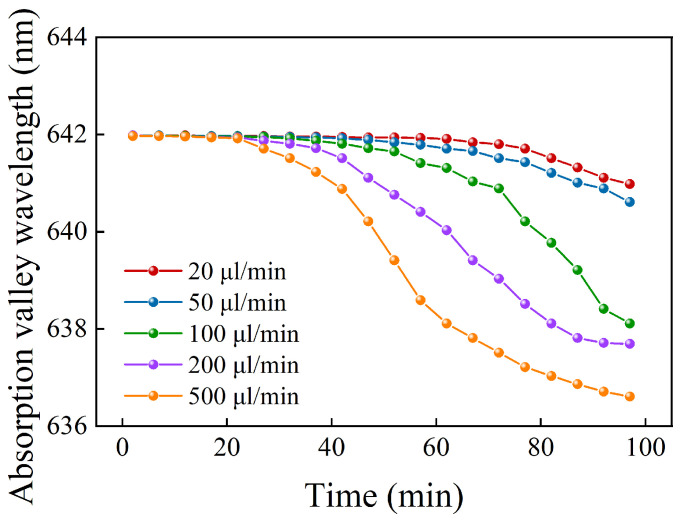
Resonance wavelength signals over time at different flow rates.

Our sensing is achieved through microfluidics, and there is a flow channel on the surface of the sensor. The actual flow rate we used was 20 µL/min. Figure 7 shows the change in the resonant wavelength signal over time at different flow rates for the optimized sensor when the detection medium is water. It can be seen that, at the flow rate we used, the sensor can be continuously used for 97 min, with a resonant wavelength shift of only 1.36 nm. This indicates that, at a safe flow rate, the sensor’s lifespan is guaranteed. However, it is undeniable that, at higher flow rates, the issue of the sensor’s lack of permanent adhesion will have some impact.

**Figure 8:**
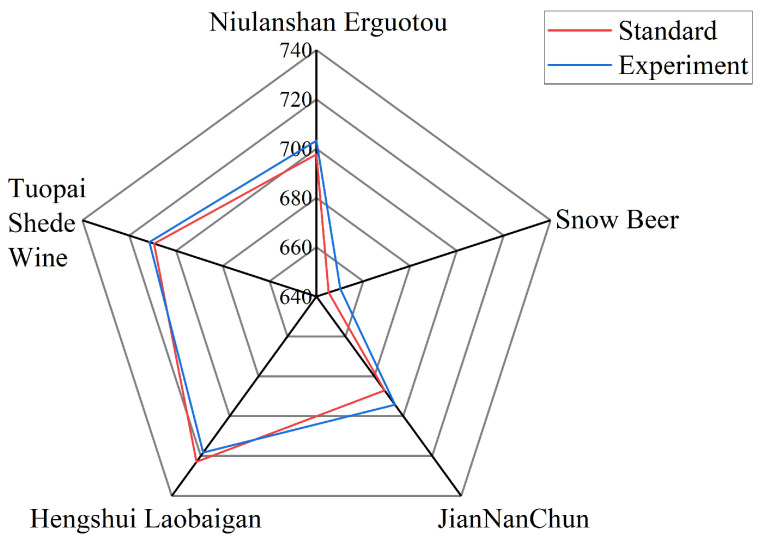
Sensing tests of the sensor on five types of alcoholic beverages.

As an application case, the proposed sensor was used to detect five common types of alcoholic beverages, as shown in Figure 8. In the experiment, the standard refractive index of an alcoholic beverage with an ethanol tolerance content (with a standard concentration) was deduced by Equation (4), the position of its standard formant was deduced by the formula in Figure 5d, and the position of its standard formant was compared with the resonant wavelength measured in the experiment. The results show good agreement between the standard values and the test values, demonstrating the potential of the proposed sensor in food detection applications.

## 5. Conclusions

In this study, a novel high-sensitivity SPR refractive index sensor based on Au-Fe_2_O_3_ nanofilm was introduced and used for alcohol detection. The synthesis method of α-Fe_2_O_3_ was introduced, and the product was characterized. The significant advantages of the nano Fe_2_O_3_ film structure with two spin-coating cycles on the SPR sensing sensitivity based on the gold film were obtained through the control experiment. The sensitivity of the sensor is 2933.2 nm/RIU, which is 30.26%, 15.01%, and 16.30% higher than that of the pure Au structure, nano-Fe_2_O_3_ film structure with a single spin-coating cycle, and nano-Fe_2_O_3_ film structure with three spin-coating cycles, respectively. In addition, the FEM simulation shows that the electric field mode increases with the increase in the coating, which confirms the experimental phenomenon. Finally, the device was used to detect the alcohol concentration in several common alcoholic beverages, showing the potential application and broad prospects of this sensor in food processing and other industries.

## Figures and Tables

**Table 1:** Comparison of the performance metrics of the sensor in this study against those from other similar studies.

Structure Details	Sensitivity to the Refractive Index	Sensitivity to Concentration	Ref.
WS_2_/Au	2459 nm/RIU	-	[33]
TiO_2_/Au	2230.9 nm/RIU	1.43 nm/%	[34]
ZnO/Au	2857 nm/RIU	1.27 nm/%	[18]
Fe_2_O_3_/Au	2933.2 nm/RIU	1.36 nm/%	This work

## Data Availability

Data are contained within the article.
